# Age-related gait adaptations: analysis of temporal gait parameters and variability, and muscle activation across flat vs. uneven surfaces in young, middle-aged, and older adults

**DOI:** 10.3389/fragi.2025.1573778

**Published:** 2025-07-24

**Authors:** Thomas B. Inns, Ilaria Pina, Lewis James Macgregor, Paul A. Dudchenko, Rachel A. Crockett, Angus Murray Hunter

**Affiliations:** ^1^ Department of Sport Science, Nottingham Trent University, Nottingham, United Kingdom; ^2^ Faculty of Natural Sciences, University of Stirling, Stirling, United Kingdom; ^3^ Faculty of Health Sciences and Sport, University of Stirling, Stirling, United Kingdom; ^4^ School of Medicine, University of Aberdeen, Aberdeen, Scotland, United Kingdom

**Keywords:** gait, ageing, neuromuscular, uneven surfaces, walking

## Abstract

**Introduction:**

Walking is a common physical activity among older adults, but it becomes more complex with age due to increased demands on motor control and attention, particularly when walking surface is uneven. Age-related changes in walking gait, especially after 70, can lead to restricted mobility and higher mortality risk. This study investigated age-related differences in gait parameters and muscle activation across young (18–39 years), middle-aged (40–59 years), and older adults (60+ years) on flat and uneven surfaces.

**Methods:**

Eighty-three healthy adults participated in the study. Variability in double support, stance, swing, and stride times; and muscle activity were assessed during walking on both surface types and used as dependent variables.

**Results:**

Analysis showed that older adults adopted a more cautious walking strategy, characterized by longer double support and shorter swing phases, regardless of the surface. Muscle activation patterns indicated increased demands on the plantar flexors and knee extensors in older adults. Walking on uneven surfaces increased gait variability across all age groups.

**Conclusion:**

These findings highlight age-specific walking adaptations related to muscle activation. Understanding these adaptations is crucial for developing targeted interventions to enhance walking performance and reduce fall risks, especially in older adults. The study underscores the importance of assessing gait under various conditions to comprehensively capture age-related differences.

## Introduction

Walking, a vital physical activity, is particularly significant for older adults engaged in ambulatory tasks (Szanton et al., 2015). Despite its perceived simplicity, walking in older adults demands heightened attention and motor control, leading to observable gait changes, notably beyond the age of 70 years (Bridenbaugh and Kressig, 2011; Ciprandi et al., 2017). These changes can impact mobility and walking performance, established risk factors for loss of independence and mortality (Hausdorff et al., 2001; White et al., 2013). Age-related alterations in walking performance are intertwined with musculoskeletal health (Schmitz et al., 2009) and motor control (Godde and Voelcker-Rehage, 2017), as reductions in muscle mass and strength, range of motion, and proprioception are compounded by deteriorating neural control, including slower motor unit recruitment, reduced firing rates, and impaired integration of sensory feedback (Power et al., 2013; Hortobágyi et al., 2015). These physiological changes disrupt coordination, stability, and adaptive gait responses. Collectively, these factors emphasize the importance of assessing gait parameters for understanding age-related changes (Salzman, 2010). It seems that subtle declines in walking performance may actually begin earlier in life, during middle age (Studenski et al., 2011; Ko et al., 2010). However, middle-aged adults remain relatively understudied in gait research compared to older cohorts, which limits our understanding of the early neuromuscular and biomechanical adaptations that may signal the onset of age-related gait deterioration.

Older adults exhibit various spatiotemporal changes in walking, including lower speed, reduced cadence, reduced stride length, and elongated double support phase (Herssens et al., 2018). Muscle activation patterns also shift, with greater coactivation of ankle and knee muscles during mid-stance and reduced soleus reliance during push-off (Schmitz et al., 2009; Vernooij et al., 2016). Increased muscle activation is associated with elevated effort and muscular demand (Kovacs, 2005) impacting daily physical activity in older adults (LaRoche et al., 2018). Precision in identifying age-related neuromuscular changes is crucial, especially in challenging situations (Kovacs, 2005), such as walking on uneven surfaces, which induces reduced speed and increased variability in older adults (da Silva Costa et al., 2020; Menant et al., 2009), introducing unpredictable perturbations that require greater neuromuscular control, sensory feedback integration, and rapid postural adjustments (Voloshina et al., 2013). Increased variability in step length, width, and timing is associated with instability, and may act as a predictor of fall risk (Hausdorff, 2005; Brach et al., 2005). In community mobility, the unevenness of surfaces is typically unpredictable (Patla and Shumway-Cook, 1999) exacerbating these challenges, yet much of our current understanding of walking perturbations associated with uneven surfaces has arisen from the study of treadmill walking (Schmitt et al., 2021). Treadmill walking is typically associated with greater cadence and shorter stride length than overground walking (Vickery-Howe et al., 2023) as well as distinct muscle activation patterns (Song and Hidler, 2008). Given these inherent differences in walking gait between treadmill and overground walking (Lee et al., 2019), there is a need for ecologically valid investigations conducted under free-walking conditions. It is challenging to assess free-walking gait under truly unpredictable conditions, previously. Okubo et al. (2019) adopted a protocol using concealed perturbations on a 10 m walkway; to ensure usual step length and cadence, participants matched their strides to a series of targets positioned on the walkway and kept time to a metronome beat, meaning that gait was not truly natural.

This study addresses gaps in our understanding of age-related differences in muscle activity and temporal gait parameters between even and uneven, unpredictable surfaces, specifically comparing young, older, and middle-aged adults. We anticipate that our findings may provide foundational insights for future interventions in community mobility. We hypothesize that older adults will exhibit maladaptations in gait parameters, showing a conservative pattern with a longer double support phase and a shorter swing phase. Additionally, we anticipate greater knee and ankle muscle activation, distinguishing older adults' gait patterns from young adults.

## Methods

### Participants and approvals

Eighty-three healthy adults aged 18–82 years provided written informed consent to take part in this study. Participants were recruited from local communities, partner organisations and contacts including churches and older adults’ fitness classes. All participants were self-reportedly physically active (exercising minimum once week), able to walk without aids, had no recent injury or surgery in the lower limbs and were free from medication, drugs, and alcohol use. All participants had normal foot posture and no postural deformities observed during the walking tasks. Individuals were split into three groups based on age: young adults 18–39 years, middle-aged 40–59 years, older adults 60+ years. Using G*Power (version 3.1.9.4), t-test difference between two independent groups (young vs. old) for double support time from Menant et al. (2009), sample size for two groups at 96% power was 24, therefore as we were including a middle aged group alongside young and old we considered 83 adults would be more than sufficient. The study was approved by the local research ethics committee and conducted according to the Declaration of Helsinki.

### Study design

For this observational cross-sectional study, participants attended a single session at the University’s Physiology Laboratory. Gait and muscular parameters, the key dependent variables for the study, were measured during a series of four continuous 4-min walking trials, two on a flat surface and two on an uneven surface. A duration of 4 min was selected to allow that a minimum of 100 strides could be completed during each trial (Riva et al., 2014). The order of trials was randomized for each participant, randomization was created through Sealed Envelope™, because of the nature of the surface conditions, it was not possible for participants to be blinded. Temporal gait parameters were assessed through in-shoe pedobarographic recordings, captured from a sample of 20 consecutive gait cycles within each 4-min trial - where a single gait cycle was identified as heel-strike to heel-strike on the dominant limb; walking speed was calculated based on linear walking to exclude periods of acceleration and deceleration; and lower limb muscle activity was determined through measurement of surface electromyography (sEMG).

### Procedures

**FIGURE 1:**
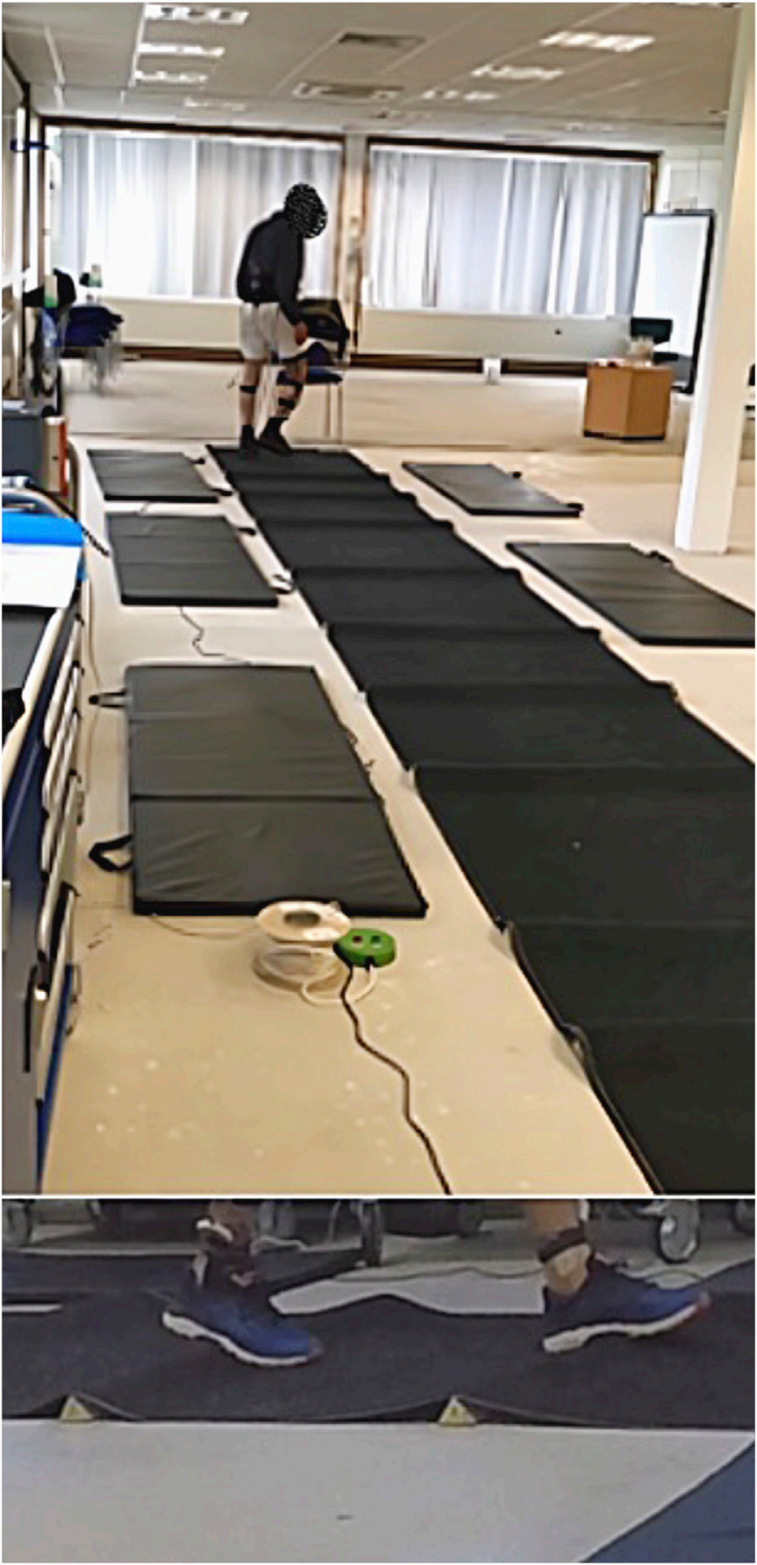
10 m uneven walking surface with 10 wooden prisms placed under the carpet randomly but with a minimum distance of 30 cm.

Upon arrival, participants’ anthropometric data were collected wearing light-weight clothing and without shoes: body mass was measured using a digital scale (Soehnle Connect, Soehnle-Waagen GmbH and Co. KG, Murrhardt, Germany) and height was assessed using a portable stadiometer (Seca 213, Birmingham, UK). Measurements were performed in duplicate, with the mean value recorded, according to International Society for the Advancement of Kinanthropometry (ISAK) guidelines, and body mass index (kg/m^2^) was calculated (World Health Organization, 1988). Trials were completed on the same 10 m carpeted track, overlaid on a concrete floor. During flat surface trials, participants walked on the track in a straight line, and were instructed to adopt their usual walking pace and keep their eyes facing forward. There was a rest period of 1 min between each trial, during which the walking surface was reconfigured as required depending on randomization. Participants were permitted to sit and rest at either end of the walking track if they felt fatigued, and were informed that the time would be paused during any rest periods - it should be noted that no rest periods were taken by any participants. Gait speed was determined by recording time taken to travel 6 m distance (between 2 m and 8 m on the track). Time recordings were captured via pressure pads positioned beneath the carpet. Speed over 6 m was measured to avoid influences of acceleration and deceleration at each end of the track (Duncan et al., 2017). Upon reaching the end of the track, participants turned 180° and continued walking until 4 min had elapsed. No more than one complete gait cycle occurred during the turning process, before linear walking recommenced. Each 180° change of direction was included in the temporal gait parameter analysis to reflect real-life walking, where change of direction is commonplace, but were excluded for the purposes of gait speed calculation and sEMG analysis. During the two randomised trials on an uneven surface participants completed the same task, with the same instructions, however the track surface was reconfigured by positioning a series of ten wooden prisms (4.0 × 4.6 × 100.0 cm) beneath the carpet at random with a minimum of 30 cm between each prism (Figure 1). Prisms were positioned at random to ensure that the uneven surface was unpredictable, to reflect the uncertainty associated with natural uneven surfaces (Patla and Shumway-Cook, 1999) and to prevent participants adopting an altered stride pattern that would have allowed them to deliberately avoid each prism (Kent et al., 2019). Since individuals have unique stride lengths and frequencies, we considered it more objective to randomly configure the uneven surface between participants, rather than providing each participant with identical surfaces, which would have resulted in relative differences (Supplementary 1).

### Pedobarography

**FIGURE 2:**
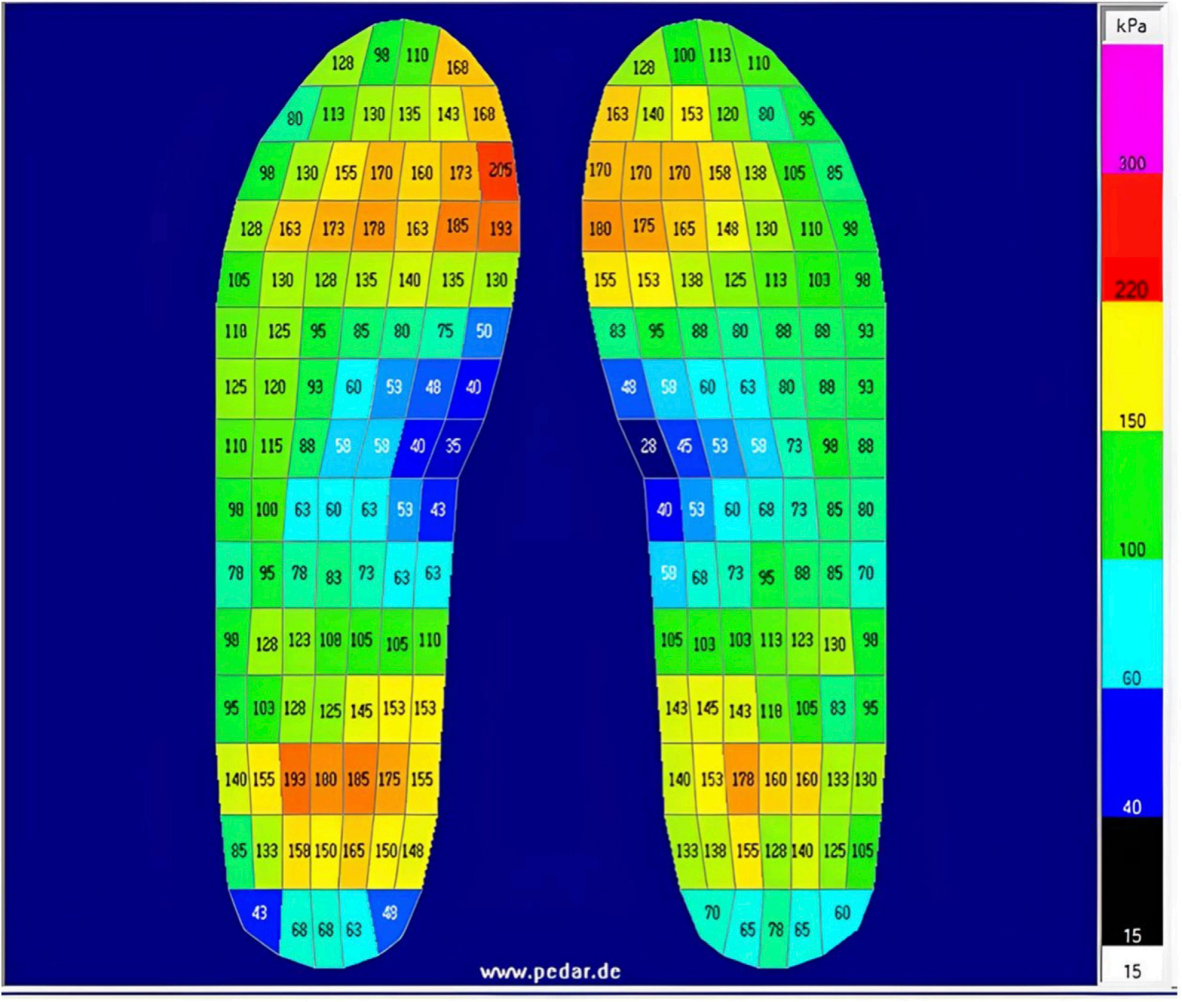
Example of average pressure detected by each sensor in the pedar-x insoles, taken from one participant during walking on the flat surface (average pressure across the 4-min trial).

**FIGURE 3:**
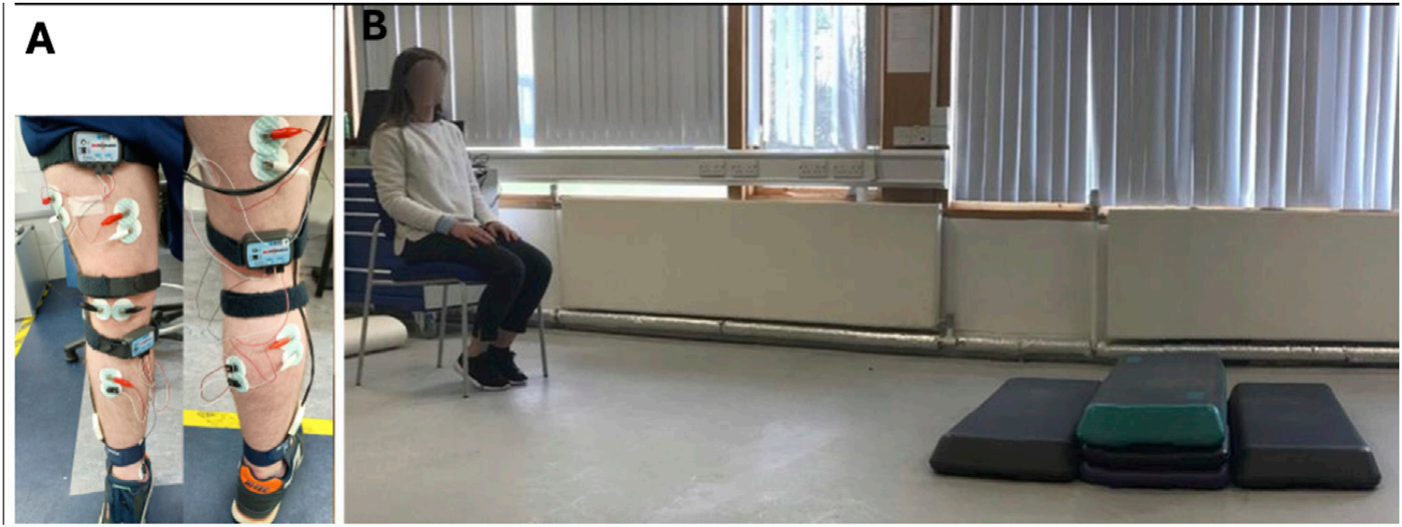
Bipolar surface electrode placement to record muscle activity **(A)**. BF: biceps femoris; VL: vastus lateralis; VM: vastus medialis; GL: lateral gastrocnemius; MG: medial gastrocnemius; and TA: tibialis anterior. An activities of daily living task was completed to provide peak surface electromyography for normalisation: standing from a chair, waling 2 m, climbing then descending two steps, walking a further 2 m before turning 180° and returning to the chair across the steps again **(B)**.

Participants wore their own flat-soled walking shoes. Pressure-sensing insoles (pedar-x, Novel GmbH, Munich, Germany) were fitted into the participants’ shoes by the researcher and remained in place until all four trials had been completed. Four insole sizes were available (EU sizes): 36/37, 38/39, 40/41, and 42/43; the largest insole that could be housed flat in the shoe was selected. Each insole contains 99 pressure-sensitive capacitive sensors distributed in a grid to cover the entire surface - Figure 2 depicts an example of pedar-x output from one participant, average pressure detected by each sensor - based on changes in capacitance, as the distance between two conductive plates changes proportionally to applied force - is displayed in kPa (range 15.0–600.0 kPa). Since sensors can be subject to degradation and/or drift over time, due to mechanical wear, repeated use, and temperature changes, insoles were calibrated before each trial, according to manufacturer guidelines. Briefly, each insole was calibrated by loading (participant standing) and unloading (participant’s foot raised off the floor). Additionally, before each data collection session, insoles were checked using the trublu^®^ calibration system, which provides a known homogenous pressure across the insole surface. Data were sampled at 50 Hz using Pedar-x software (Novel GmbH, Munich, Germany). The gait analysis software programme (Pedar Online Novel GmbH, Munich, Germany) was used to identify the first and second heel contacts during usual speed walking on both surface conditions, and these heel contact events were used to locate gait cycle alongside sEMG onset/offset timings. The insoles offer detailed topographic pressure maps, capturing fine pressure gradients across the foot continuously over time, allowing analysis of specific gait phases. Although it was beyond the scope of this study, data can be represented as 2D (e.g., Figure 2) or 3D pressure maps, showing both magnitude and location of peak pressures at each phase, allowing analysis of temporal pressure changes across the gait cycle. From each 4-min trial, the same number of consecutive gait cycles (20 consecutive cycles) were identified for each participant and each trial, these were included in the temporal gait parameter analysis. These series of 20 consecutive cycles were visually inspected before selection to ensure that no observable outliers were included. Four minutes of continuous walking (⩾100 strides) should be sufficient to allow isolation of 20 consecutive gait cycles that are reliably representative of normal walking gait (Hollman et al., 2010; Kribus-Shmiel et al., 2018; Kroneberg et al., 2019). Total stride time (heel-strike to heel-strike on the dominant limb), and time spent in stance (from heel-strike to toe-off) and in swing (from toe-off to heel-strike) were recorded from each gait cycle. Gait variability, defined as the variations that occur across multiple strides (Hausdorff, 2005), was also measured. Variability was measured with coefficient of variation (CV). The CV values were calculated using the formula: (σ/μ)*100; where σ is the standard deviation, and μ is the mean of the 20 included gait cycles (Hausdorff, 2005). A number of temporal pedobarographic parameters can also be calculated by the system, the most clinically relevant of these have previously been reported to be contact area, contact time in percentage roll over process, maximum force, pressure–time integral, force–time integral, peak pressure, mean force, and mean area; reliability of these parameters has also been reported elsewhere (Putti et al., 2007; Ramanathan et al., 2010).

### Muscle activity

sEMG was captured using wireless sensors (Biopac Systems Inc, Goleta US). Prior to electrode placement, skin was prepared according to SENIAM recommendations (Stegman and Hermans, 2007). A pair of self-adhesive Ag/AgCl disc electrodes (36 × 40 mm) (PNS Dual Element Electrode; Vermed, Vermont, USA) were placed on the skin over six muscle sites of the right lower limb (Figure 3A): biceps femoris (BF), vastus lateralis (VL), vastus medialis (VM), lateral gastrocnemius (GL), medial gastrocnemius (GM) and tibialis anterior (TA). The interelectrode distance was 20 mm. All sites were located according to the Anatomical Guide for the Electromyographer (Murray et al., 1995). These muscles perform fundamental roles in mobility and were selected because their weakness has been associated with postural instability and increased risk of falls in older adults (Horlings et al., 2008; Horlings et al., 2008). Reference electrodes were positioned over the patella (BF, VL, VM) and medial malleolus (GM, GL, TA). Signals were sampled at 2000 Hz using Acqknowledge software (version 3.9.1, BIOPAC Systems Inc, Goleta US), bandpass filtered (20–300 Hz) (Martinek et al., 2021), and notch-filtered at 60 Hz using a Butterworth filter. Our optimal signal to noise ratio was considered to be above 20dB, our pre amplification was set at a gain of 5,000 unless the signal was clipped therefore, we would then drop to 2000. Before the first trial, to provide a peak dynamic sEMG recording to which subsequent sEMG signals were normalised, participants performed an activities of daily living task, as described elsewhere (Ghazwan et al., 2017). Briefly, participants began seated on a straight-backed chair, before standing unaided, walking 2 m and ascending then descending two 10 cm steps. Participants then walked for a further 2 m, before turning 180°, and returning to the chair via the same path; the task ended when the participant sat back down on the chair (Figure 3B). Signals captured during each 4-min trial were root-mean square (RMS) processed with window length 200 m and normalised to the peak dynamic amplitude recorded during the activities of daily living task. This approach for normalising sEMG during walking has been shown to provide a valid reflection of the relative magnitude of muscle activity and of onset/offset timings (Hollman et al., 2010). sEMG signals were synchronized with temporal gait parameters offline, by down-sampling to 50 Hz and EMG cycles were time-normalised to 101 points from 0 to 100 (MS Excel). Each complete gait cycle was divided into phases based on previous literature (Bailey et al., 2019): loading (0%–10%), mid-stance (10%–30%), terminal stance (30%–60%), initial swing (60%–73%), mid-swing (73%–87%), and terminal swing (87%–100%).

### Statistical analysis

Statistical analysis was performed using GraphPad Prism for Windows, version 10 (GraphPad Software, MA, USA). Descriptive statistics for continuous variables were expressed as mean and standard deviation (SD) or median and interquartile range (IQR) as appropriate. Univariate analyses were performed to assess differences between demographic variables, with one-way Analysis of Variance (ANOVA) and Kruskal–Wallis tests used for parametric and non-parametric data respectively as required. Normality was assessed through the Shapiro-Wilk Test and visual assessment through the Q-Q plot. To test hypotheses examining the effects of age and surface on gait temporal parameters and muscle, 2-way repeated measures ANOVA were performed using age (young/middle/older adults) as a between-subject factor and surface (flat/uneven) as a within-subject factor. Planned comparisons were done with Independent sample T-tests for each age group to assess the strength of any main effects identified in the ANOVA tests. Partial Eta-Squared (η_p_^2^) was used to quantify effect sizes. Separate analyses were performed for each dependent variable (temporal parameters and muscle activation). In the case of a significant interactions or main effects, *post hoc* multiple comparison pairwise analysis utilising Bonferroni corrections were used to compare differences in groups. A *p*-value of ⩽0.05 was considered as statistically significant.

## Results

### Participant characteristics

No significant differences were present in demographic variables across the three age groups (Table 1). No significant differences were found across the age groups for normal gait speed.

**TABLE 1:** Descriptive variables reported as mean ± SD (normally distributed) or median (IQR, 25th-75th percentile) (skewed distribution).

Variable	18-39y young adults (n = 31)	40-59y middle-aged adults (n = 17)	60+ y older adults (n = 35)
Age (years)	**26.5 ± 5.05**	**48.4 ± 6.3**	**68.9 ± 6.2**
Sex (n, %), Female	17 (54.8)	13 (76.5)	21 (60.0)
Dominant leg (n, %), left	3 (9.7)	3 (17.6)	1 (2.9)
Body mass (kg)	67.0 (58.0–84.7)	65.0 (56.5–82.65)	72.0 (64.0–88.0)
Height (cm)	169.9 ± 10.7	166.5 ± 11.9	168.1 ± 9.4
Body mass index (kg/m^2^)	23.5 (22.1–25.8)	25.4 (22.1–28.4)	25.8 (22.7–29.4)
Gait speed (m/s)	1.05 (0.97–1.14)	1.05 (0.99–1.07)	1.03 (0.98–1.10)

One-way Analysis of Variance and Kruskal–Wallis tests were conducted on normally distributed and skewed data, respectively. Statistically significant differences between groups (p ⩽ 0.05) are highlighted in bold.

### Temporal gait parameters

#### Stride, stance, and swing time

Stride time was significantly longer on the uneven surface compared with flat surface (*F*_(1,78)_ = 24.20, *p* < 0.01, η_p_^2^ = 0.236) (Table 2). This effect was seen in all age groups: young (uneven: 1.37 ± 0.16 vs. flat 1.29 ± 0.15; t (78) = 2.74, *p* = 0.023), middle-aged (uneven: 1.42 ± 0.48 vs. flat: 1.30 ± 0.25; t (78) = 2.91, *p* = 0.014) and older adults (uneven: 1.28 ± 0.18 vs. flat: 1.20 ± 0.16; t (78) = 3.00 *p* = 0.011).

**TABLE 2:** Stride, stance and swing phase times for each age group and surface type reported as mean ± SD. Symbols indicate significantly greater time than the alternate surface. Significant differences between surface type within age group denoted as follows: * = p < 0.05, ** = p < 0.01, *** = p < 0.001.

Gait phase	Surface type	18-39y young adults (n = 31)	40-59y middle-aged adults (n = 17)	60+ y older adults (n = 35)
Stride time (s)	Flat	1.28 ± 0.15	1.30 ± 0.25	1.20 ± 0.16
	Uneven	1.37 ± 0.16*	1.42 ± 0.48*	1.28 ± 0.18*
Stance time (s)	Flat	0.79 ± 0.08	0.77 ± 0.08	0.77 ± 0.15
	Uneven	0.84 ± 0.10	0.83 ± 0.14	0.84 ± 0.22**
	Δ% of stride	−1%	+1%	+1%
Swing time (s)	Flat	0.49 ± 0.05	0.46 ± 0.06	0.43 ± 0.06
	Uneven	0.53 ± 0.05***	**0.48 ± 0.08**	0.46 ± 0.09*
	Δ% of stride	+1%	−1%	0%

For stance time, significantly longer durations were observed on the uneven surface compared with the flat surface (*F*_(1,80)_ = 18.88, *p* < 0.01, η_p_^2^ = 0.191). This difference, however, only reached significance for older adults (uneven: 0.84 ± 0.22 vs. flat: 0.77 ± 0.15; t (80) = 3.30, *p* < 0.01).

Significantly longer swing times were observed on the uneven surface compared to the flat surface (*F*_(1,80)_ = 21.18, *p* < 0.01, η_p_^2^ = 0.209). Planned comparison revealed that longer swing time durations were seen in the young group (uneven: 0.53 ± 0.05 vs. flat: 0.49 ± 0.05; t (80) = 3.80, *p* < 0.01) and the older group (uneven: 0.46 ± 0.09 vs. flat: 0.43 ± 0.07; t (80) = 2.70, *p* = 0.025). A significant main effect of age was also present (*F*_(2,80)_ = 9.850, *p* < 0.01, η_p_^2^ = 0.514). This was reflected by a longer swing time in young adults compared to older adults (t (80) = 4.43, *p* < 0.01).

### Gait phases variability

**FIGURE 4:**
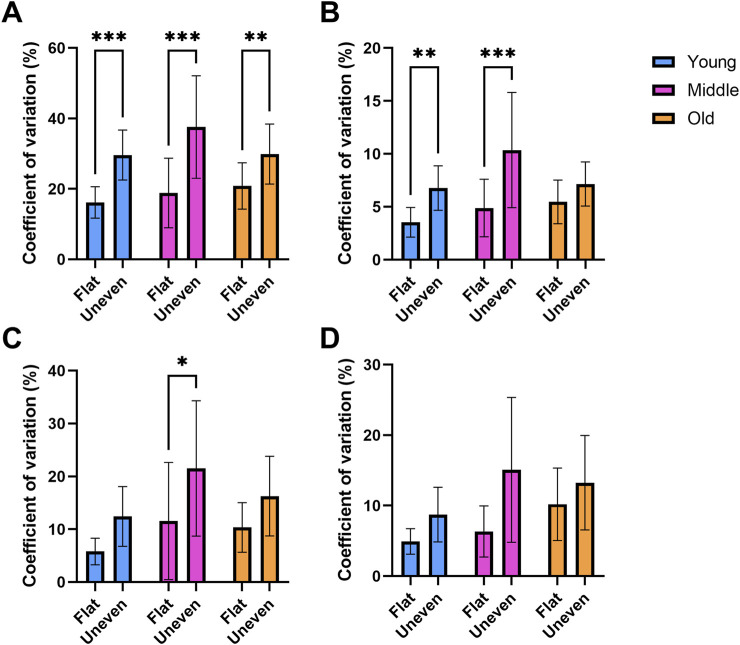
Double support (**(A)** n = 79), stance (**(B)** n = 81), swing (**(C)** n = 81) and stride (**(D)** n = 80) times coefficient of variation recorded while walking across flat and uneven surfaces between young, middle aged and older adult groups. * = p < 0.05, ** = p < 0.01, *** = p < 0.001.

A significant main effect of surface was observed for double support variability (*F*_(1,78)_ = 47.5, *p* < 0.01, η_p_^2^ = 0.378, Figure 4A), with young (t (78) = 4.34, *p* < 0.01), middle-aged (t (78) = 4.41, *p* < 0.01) and older adults (t (78) = 3.16, *p* < 0.01) expressing greater variability on the uneven surface compared to the flat surface.

A significant main effect of surface for stance phase variability (*F*_(1,78)_ = 25.7, *p* < 0.01, η_p_^2^ = 0.248, Figure 4B) was observed, which was reflected by a greater degree of variability on the uneven surface compared to the flat surface in the young (t (78 = 3.04, *p* = 0.01) and middle-aged adults (t (78) = 3.76, *p* < 0.01).

For swing phase variability, there was a significant difference between surfaces (*F*_(1,78)_ = 15.9, *p* < 0.01, η_p_^2^ = 0.170, Figure 4C). Middle-aged adults expressed a greater variability on the uneven surface vs. flat surface (t (78) = 2.49, *p* = 0.045).

A significant main effect of surface (*F*_(1,78)_ = 7.94, *p* < 0.01, η_p_^2^ = 0.093, Figure 4D) was also found for stride time variability (uneven: 14.96% ± 31.94% vs. flat: 7.35% ± 10.72%). However, the direction of this effect cannot be confirmed because no significant *post hoc* tests were observed between age groups.

### Muscle activation

#### Loading

**FIGURE 5:**
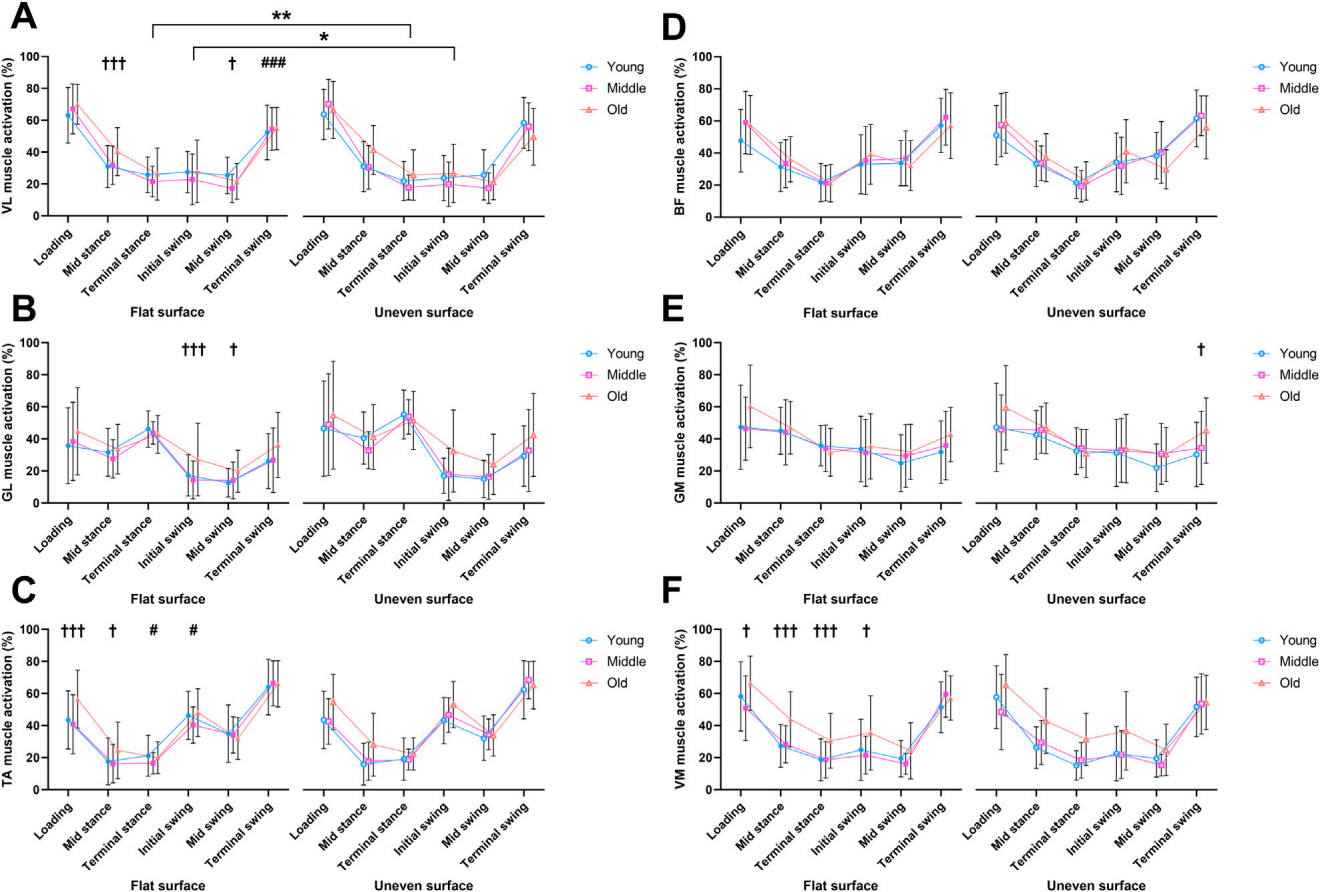
Mean muscle activation percentages measured across the phases of the gait cycle in flat and uneven walking trials between young, middle-aged and older adult groups. Vastus lateralis **(A)**, Gastrocnemius lateralis **(B)**, Tibialis anterior **(C)**, Biceps femoris **(D)**, Gastrocnemius medialis **(E)**, and Vastus medialis **(F)** muscles. † = significant main effect of age, * = significant effect of surface, # = significant interaction. * = p < 0.05, ** = p < 0.01, *** = p < 0.001.

There was a significant main effect of age for the TA during the loading phase (*F*_(2,77)_ = 6.19, *p* < 0.01, η_p_^2^ = 0.467), with *post hoc* testing showing older adults having greater activation during this phase vs. young (t (77) = 2.98, *p* = 0.012) and middle-aged (t (77) = 2.86, *p* = 0.017) adults (Figure 5C). No differences were found between young and middle-aged adults (t (77) = 0.365, *p* > 0.999).

There was also a significant main effect of age for the VM during the loading phase (*F*_(2,77)_ = 4.29, *p* = 0.017, η_p_^2^ = 0.462) which was reflected by a greater activation in older adults compared to middle-aged adults (t (77) = 2.86, *p* = 0.016) (Figure 5F).

No significant changes were found during the loading phase for the VL, GM, GL or BF (Figures 5A,B,D,E).

#### Mid-stance, terminal stance, and initial swing

During the mid-stance phase, there were significant main effects of age for the VL (*F*_(2,77)_ = 5.09, *p* < 0.01, η_p_^2^ = 0.478), with older adults displaying greater activation compared to young adults (t (77) = 2.87, *p* = 0.016, Figure 5A). This was also the case in the TA, with a main effect of age (*F*_(2,77)_ = 3.82, *p* = 0.026, η_p_^2^ = 0.507) reflecting greater activation in older compared to young adults (t (77) = 2.50, *p* = 0.044, Figure 5C). A significant main effect of age was seen in the VM (*F*_(2,77)_ = 10.9, *p* < 0.01, η_p_^2^ = 0.758), with older adults expressing greater activation than both young (t (77) = 4.36, *p* < 0.01) and middle-aged adults (t (77) = 3.18, *p* < 0.01, Figure 5F). No significant effects were observed in the GL, GM, or BF for the mid-stance phase (Figures 5B,D,E).

During the terminal stance phase, there was a significant main effect of surface in the activation of the VL (*F*_(1,77)_ = 6.12, *p* = 0.016, η_p_^2^ = 0.074), however no significant *post hoc* tests were observed (Figure 5A). For the TA, there was a significant interaction (*F*_(2,77)_ = 3.95, *p* = 0.023, η_p_^2^ = 0.093), although no significant *post hoc* tests were observed between groups (Figure 5C). A significant main effect of age was observed in the VM (*F*_(2,77)_ = 10.4, *p* = 0.013, η_p_^2^ = 0.681) with older adults expressing greater activation than both young (t (77) = 4.25, *p* < 0.01) and middle-aged adults (t (77) = 3.14, *p* < 0.01, Figure 5F). No significant effects were observed in the GL, GM, or BF for the terminal stance phase (Figures 5B,D,E).

During the initial swing phase, a significant main effect of surface was present for VL activation (*F*_(1,77)_ = 4.50, *p* = 0.037, η_p_^2^ = 0.055), although no significant *post hoc* tests were observed (Figure 5A). For the GL, a main effect of age was present (*F*_(2,77)_ = 5.01, *p* < 0.01, η_p_^2^ = 0.655), with older adults expressing greater activation than both young (t (77) = 2.74, *p* 0.025) and middle-aged adults (t (77) = 2.50, *p* = 0.044, Figure 5B). A significant interaction was observed in the TA (*F*_(2,77)_ = 4.74, *p* = 0.011, η_p_^2^ = 0.110), with older adults expressing a greater activation on the uneven surface than young adults (t (154) = 2.83, Figure 5C). While no significant changes were observed in the BF and GM (Figures 5D,E), a significant main effect of age was observed in the VM (*F*_(2,77)_ = 4.57, *p* = 0.013, η_p_^2^ = 0.596, Figure 5F). This was reflected by older adults expressing greater activation than both young (t (77) = 2.55, *p* = 0.038) and middle-aged adults (t (77) = 2.46, *p* = 0.048).

#### Mid-swing and terminal swing

For the mid-swing phase, a significant main effect of age was observed in the VL (*F*_(2,77)_ = 3.19, *p* = 0.047, η_p_^2^ = 0.278), with young adults expressing greater VL activation than middle-aged adults (t (77) = 2.48, *p* = 0.046, Figure 5A). A significant main effect of age was also found for the GL (*F*_(2,77)_ = 3.78, *p* = 0.027, η_p_^2^ = 0.390) reflected by older adults expressing greater activation than young adults (t (77) = 2.61, *p* = 0.033, Figure 5B). No significant effects were observed during the mid-swing phase for the TA, BF, GM, or VM (Figures 5C–F).

During the terminal swing phase, there was a significant interaction for VL muscle activation (*F*_(2,77)_ = 5.06, *p* < 0.01, η_p_^2^ = 0.116, Figure 5A). However, this was not accompanied by any significant *post hoc* tests between groups. A significant main effect of age was observed for the GM (*F*_(2,77)_ = 3.96, *p* = 0.023, η_p_^2^ = 0.473), with older adults expressing greater activation than younger adults (t (77) = 2.76, *p* = 0.022, Figure 5E). For the GL, TA, BF, and VM, there were no significant differences observed during the terminal swing phase (Figures 5B–D,F).

## Discussion

In this study, we compared walking gait and skeletal muscle recruitment among young, middle-aged, and older adults on even and uneven, unpredictable surfaces. Uneven surfaces necessitated a more cautious walking strategy for all age groups, characterized by longer stride time, but older adults appear to navigate these more-challenging environments with reliance on greater muscle activation (Maki, 1997). While all age groups displayed slower stride time on uneven surfaces, it was only among older adults that both stance and swing time were seen to be prolonged; this is indicative of an overall slowing of the gait cycle as opposed to a proportional prolonging of stance phase relative to swing phase. Such a cautious approach is likely aimed at increasing stability by reducing time spent weight-bearing on a single leg to counter age-related declines in lower limb muscle strength (Aboutorabi et al., 2016). These findings align with previous literature, where age-related changes have been interpreted as a shift towards a more conservative gait, as has been reported on uneven surfaces requiring deliberate obstacle avoidance (Rosengren et al., 1998).

Temporal gait parameter variability, a predictor of falls (Hausdorff et al., 2001), revealed an irregular gait pattern on uneven surfaces across all ages. The pronounced differences on uneven surfaces indicate that older individuals may perceive the unevenness and/or unpredictability of these surfaces as a greater stability threat, leading to an even more conservative gait pattern than on flat surfaces (Dixon et al., 2018; Marigold and Patla, 2008). Walking gait has been shown to be impacted by age-related changes in neuromuscular function, such as reductions in motor unit discharge rates, altered recruitment patterns due to motor unit loss, and increased motor unit synchronisation (Clark et al., 2012; Power et al., 2013). These changes contribute to diminished force output and neuromuscular precision, which may result in the compensatory cautious strategy we have observed (Hortobágyi et al., 2015). Interestingly, adoption of a conservative gait pattern, as noted here, has previously been linked to declining muscle strength (Kent et al., 2019), which is often initiated earlier in adulthood than observable decreases in gait quality (Haynes et al., 2020). Previous studies linked a prolonged double support phase to fear of falling (Makino et al., 2017), prevalent in >20% of community-dwelling older adults (Evitt, 2004). While differences in walking could also be interpreted as resulting from increased difficulty associated navigating an uneven surface, crucially the walking task in this study did not involve obstacle avoidance (Shimizu et al., 2020). Therefore, increased caution whilst walking is a likely explanation for the observed alterations in gait. Therefore, future studies on age-related walking changes should consider psychological factors, such as fear of falling, to understand their role in altered gait patterns related to age and surface conditions.

Our findings highlighted greater muscle activation of plantar flexors, dorsiflexors, and knee extensors among older adults, supporting the concept of an age-related functional shift in power production during walking (Schloemer et al., 2017). This shift is evident in changes in the relative contribution of specific muscle groups (Schloemer et al., 2017). Higher muscle activity during walking is associated with increased effort and muscular demand (Hortobágyi et al., 2011) and is linked to reduced daily stepping time and sit-to-stand transitions in older adults (LaRoche et al., 2018). Within gait cycle co-contraction activation patterns are similar to previously reported (Neptune et al., 2004; Perry et al., 2010) although no increased co-contraction was observed in BF vs. VL in females vs. males as shown previously in 18 females and 17 males (Qi et al., 2023), however our study has far greater number of participants, therefore greater statistical power. These findings provide insights for future exercise programmes aiming to reduce walking effort and support physical activity engagement in older adults. Although we observed some walking surface-related differences in muscle activation, lack of significant *post hoc* effects limit our interpretation of these. Nonetheless, we might consider it somewhat surprising that greater between-condition variations were not detected. Previously Schrijvers et al. (2021) reported a dose dependant response, whereby greater intensity perturbations elicited greater muscle activation, and distinct activation patters were observed among some muscles in response to different types of perturbation. Our aim was to assess walking gait on an uneven, unpredictable surface - as such, we did not control the nature of perturbations that participants experienced. Throughout each trial, foot contact location on the prisms varied, such that foot-prism interaction occurred with heel, midfoot, or forefoot contact, at random. Accordingly, the degree of plantar- and dorsiflexion varied. It is possible that the mechanical demand - and therefore muscle activation - would be affected in contrasting ways within the same trial condition, perhaps obscuring between-condition differences. We propose that future research should attempt to track specific perturbation types, without allowing the walking task to become predictable.

We identified age-specific alterations in walking gait associated with physiological changes in skeletal muscle activation, potentially linked to muscle function. The adoption of a more cautious walking strategy may be attributed to natural age-related strength loss (Hahn et al., 2005). Our findings support the significance of knee extensor function in aging (Bailey et al., 2019), emphasizing the need for targeted interventions for older adults. While triceps surae neuromuscular training did not meaningfully improve response to perturbations in older women (Epro et al., 2018) multimodal interventions including resistance training are warranted to improve gait stability and in turn reduce the potential fall risk it may lead to (Zhong et al., 2024). However, it remains uncertain whether observed changes in muscle activation result from inherent aging processes or voluntary alterations influenced by psychological factors linked to fear of falling.

Importantly, we included middle-aged participants within our analysis. This group demonstrated the most pronounced difference in gait variability between flat and uneven surface, with greater variability observed during uneven surface walking in double support, stance, and swing phases. While middle age is often assumed to be functionally stable, these findings suggest that gait and neuromuscular control may begin to decline earlier than previously believed (Bohannon et al., 1997; Haynes et al., 2020). Middle age may represent a transitional phase during which subtle declines in muscle strength, coordination, and neuromuscular control accumulate and begin to influence walking behaviour (Ko et al., 2010). These changes may not yet impair overground walking across flat surfaces, but may become more apparent under challenging conditions such as uneven terrain, where adaptive capacity is taxed.

Several factors require consideration in interpreting these results. Middle-aged participants (40–59 years) exhibited higher variability in each gait phase on uneven surfaces; while comfortable speed walking gait typically remains unimpaired until beyond the age of 60 years (Zadik et al., 2022), maximum walking speed declines more rapidly with advancing age (Bohannon et al., 1997), and while other markers of skeletal muscle function - such as dynamic knee extension strength (Haynes et al., 2020) - can show declines from around 40 years of age. Middle-age might be considered to be a dynamic period during which early neuromuscular deterioration are already be underway. Such variable decline among this age group makes understanding the status of middle-aged adults more challenging and highlights need for further research into the transition from youth to older age (Lachman, 2015). We assessed age-related gait changes at participants' usual walking speed, which did not differ across age groups. Therefore, future studies should incorporate both usual and fast measurements, as fast gait speed decline over 11-year follow-up was shown to predict future disability (Artaud et al., 2015). We must acknowledge that self-selection bias could have been present, potentially limiting the generalizability of these results to middle-aged and older adults with self-perceived mobility impairments - future studies should control for personal preferences and attitudes in volunteer populations. In terms of statistical analysis, homogeneity of variance was assessed through visual inspection of residual plots. While the majority of models met the assumption, a small number did not. While repeated measures ANOVA is generally robust to moderate violations, results should be interpreted with caution. We must also consider whether the uneven surface condition adopted should be considered truly unpredictable; limitations in previous studies have included reliance on visual and auditory cues to control stride length and cadence (Okubo et al., 2019) or the use of treadmill surfaces as opposed to overground free-walking (Madigan et al., 2018). While our uneven condition represents high ecological validity, we cannot rule out the possibility that participants were able to predict upcoming perturbations, particularly towards the latter stages of each walking trail. Finally, were limited by EMG system capacity to only record one lower limb therefore recommend future studies obtain bilateral EMG to provide more insight into muscle activation patterns during varied terrain walking.

## Conclusion

In conclusion, older adults consistently demonstrated a cautious walking strategy, characterized by prolonged double support and a shorter swing phase, irrespective of surface conditions. Elevated muscle activity across various muscle groups highlighted increased skeletal muscular demand with age, further emphasized on uneven surfaces, suggesting a perceived stability threat. These findings support existing literature linking gait irregularities to fear of falling. Particularly notable are muscle activation changes in quadriceps knee extensors, providing valuable insights for targeted strength training in older adults as part of multimodal interventions. However, study limitations warrant cautious generalization, and future research should investigate the interplay between physiological and psychological factors influencing age-related walking gait changes.

## Data Availability

The raw data supporting the conclusions of this article will be made available by the authors, without undue reservation.
